# Advances in African swine fever virus molecular biology and host interactions contributing to new tools for control

**DOI:** 10.1128/jvi.00932-24

**Published:** 2025-05-09

**Authors:** Linda K. Dixon

**Affiliations:** 1 The Pirbright Institute, Pirbright, Woking, Surrey, United Kingdom; Universiteit Gent, Merelbeke, Belgium

**Keywords:** African swine fever virus, genome, replication, immune evasion, virulence factors

## Abstract

African swine fever virus (ASFV) causes a frequently fatal hemorrhagic disease in domestic pigs and wild boar. The spread from Africa to Georgia in 2007 initiated a pandemic affecting many European and most Asian countries. This has had a very high socio-economic impact and threatens global food security. The virus is a large, complex, cytoplasmic DNA virus, the only member of the *Asfarviridae* family and codes for 170–190 proteins. Many of these have unknown functions and do not resemble other viruses or host proteins. This complexity has hindered the development of vaccines and other tools for control. The intensity of research has increased since the spread of ASFV in Europe and Asia, leading to rapid advances in knowledge. This review summarizes recent research, including the determination by cryogenic electron microscopy of the virus capsid structure and virion proteome. Novel information on the virus replication cycle, including mechanisms of virus entry into cells and the identification of host endosomal proteins important for entry, is summarized. Multiple, novel virus immune evasion proteins and their targets in the type I interferon response and inflammation pathways have been identified. The potential for the application of this knowledge to developing novel control tools, including modified live vaccines and other interventions targeting critical virus processes or host interactions, is discussed.

## INTRODUCTION

The current African swine fever virus (ASFV) pandemic started in 2007 following an introduction from East Africa to Georgia in the Caucasus region of Europe. From there, the disease spread to parts of Russia and many other Eastern European countries. In 2018, disaster struck as the virus entered China, which has more than half the global pig population. This resulted in a very large loss in the production of the Chinese herd in 2019, with global knock-on effects on supplies and prices. Currently, almost all Asian countries, plus some in Oceania, are infected. In 2021, ASFV spreads to the Dominican Republic and Haiti in the Caribbean (see FAO Empres ASF situation in Asia & Pacific update, African swine fever - WOAH - World Organisation for Animal Health).

ASFV originated and has been present over many years in an ancient sylvatic cycle in East Africa involving warthogs and soft ticks, *Ornithodoros moubata*, that inhabit their burrows. In these hosts and other endemic African wild suids, few if any clinical signs are observed (1). In contrast, ASFV causes hemorrhagic fever in domestic pigs and wild boar that can result in the death of almost all infected animals. Lower virulence isolates have been described as circulating in domestic pigs or wild boar. These can cause lower or no fatality but sometimes result in a chronic form of the disease (2, 3).

ASFV is the only member of the *Asfarviridae* family in the *Nucleocytoviricota* phylum. The complexity of the large virus particle and double-stranded DNA genome coding for up to 170–190 proteins has contributed to the difficulty in developing vaccines (4). Vietnam was the first country to license the use of modified live vaccines in 2023 (5). Other factors, including the environmental stability of the virus and the presence in wildlife reservoirs, contribute to difficulties in the control of ASFV. Research efforts on ASFV have increased dramatically, particularly since 2018. Here, recent research on ASFV molecular biology and virus-host interactions is reviewed, and the contribution to increased understanding and development of new control measures is evaluated.

## VIRUS STRUCTURE

**Fig 1:**
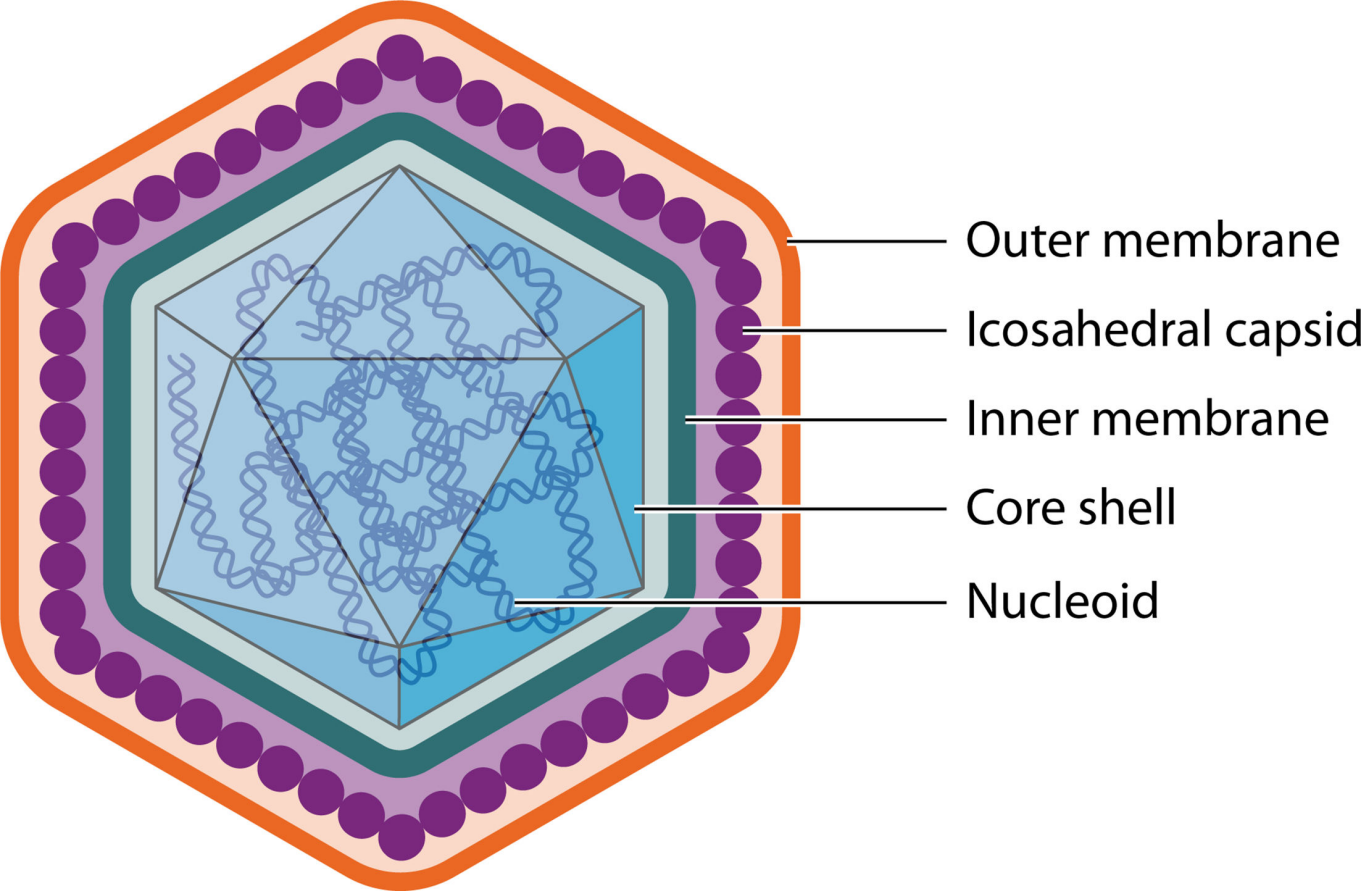
The multi-layered structure of the ASFV particle. The diagram shows the structural layers of the ASFV particle. The outer membrane is gained by budding from the host plasma membrane. The virus capsid is assembled on the inner membrane below which the core shell or inner capsid is formed. The nucleoid containing the virus genome and other proteins is inside the core shell. The capsid is 250 nm and core shell 180 nm maximum diameter (6–8).

Rapid progress has been made in determining the structure and protein content of ASFV particles. These have a large, complex structure consisting of a nucleoprotein core that contains the genome and enzymes, including the virus-encoded RNA polymerase (RNAP), along with other factors required to initiate the next round of infection. The core is surrounded by an icosahedral core protein shell, also known as the inner capsid and an inner envelope onto which the icosahedral capsid is assembled. Extracellular virions gain an additional envelope derived from the plasma membrane (see Fig. 1) (4).

Proteomic analysis of highly purified virions from Vero cells infected with the tissue culture-adapted isolate BA71V identified 68 viral proteins, including 44 that were detected in virions for the first time. Functions of virion proteins include those involved in virus architecture and others for use early during the replication cycle. Only one viral protein, pCD2v (*EP402R*), was identified in the outer envelope (9). Since this publication, additional functions have been assigned to virion proteins (see Table 1).

**TABLE 1:** ASFV proteins detected by mass spectrometry in virions (9)^*a*^

Protein	Function	Location	Transcribed
CD2v	Hemadsorption	Outer envelope	Late
B646L	p72 major capsid protein	Capsid	Late
B438L	Capsid pentomer (7, 8)	Capsid	Late
M1249L	Capsid structure and transcription (7, 8, 10, 11)	Capsid/nucleoid	Late
H240R	Penton protein and IFN response (7, 8, 12)	Capsid	Late
D117L	Transmembrane p17 and p72 membrane anchoring (7, 8)	Inner membrane	Late
CP123L	Transmembrane domain	Inner membrane	Late
E248R	Transmembrane entry fusion (13)	Inner membrane	Late
KP177R	Transmembrane domain	Inner membrane	Late
E146L	Transmembrane domain	Inner membrane	Late
E199L	Transmembrane entry fusion (14)	Inner membrane	Late
O61R	Virus attachment protein (15)	Inner membrane	Late
EP152R	Transmembrane domain	Inner membrane	Late
B117L	Transmembrane domain and viroporin (16)	Inner membrane	Late
B169L	Transmembrane domains and viroporin (17)	Inner membrane	Late
E183L/p54	Entry, formation of precursor viral membranes (18)	Inner membrane	Late
EP84R	Guides core shell proteins to inner envelope (19)	Inner membrane	Late
CP2475L	pp220 processed to p150, p34, p37, p14, and p5 (20)	Core shell	Late
CP530R	pp62 processed to p35, p15, and p8 (20)	Core shell	Late
S273R	Sumo-like protease. Polyprotein processing (21)	Core shell	Late
B962L	VACVI8-like helicase	Nucleoid	Late
D1133L	VACVD6-like helicase, early transcription	Nucleoid	Late
Q706L	VACV D11 helicase	Nucleoid	Late
K78R	p10 DNA binding	Nucleoid	Late
NP868R	mRNA capping	Nucleoid	Early
C475L	Poly A polymerase	Nucleoid	Late
NP1450L	RNAP subunit 1	Nucleoid	Early
EP1242L	RNAP subunit 2	Nucleoid	NC
D339L	RNAP subunit 7	Nucleoid	Late
C105R	RNAP 9	Nucleoid	NC
D205R	RNAP subunit 5	Nucleoid	Late
C147L	RNAP subunit 6	Nucleoid	Late
H359L	RNAP subunit 3/11	Nucleoid	Early
NP419L	DNA ligase	Nucleoid	Late
G1340L	VACV A7 early transcription factor	Nucleoid	Late
CP312R	Single-stranded DNA (ssDNA) binding and ribosome binding (22, 23)	Nucleoid	Early
K78R	p10 DNA binding	Nucleoid	Late
A104R	Histone-like DNA binding and IFN response	Nucleoid	Late
O174L	DNA polymerase X and DNA repair	Nucleoid	Late
E296R	AP endonuclease and DNA repair	Nucleoid	Early
E165R	dUTPase and DNA repair	Nucleoid	Early
EP424R	Putative RNA methylase	Unknown	Early
R298L	Serine/threonine protein kinase	Unknown	Late
A137R	IFN inhibitor, forms dodecahedron cage (24, 25)	Unknown	Late
C129R	cGAMP cleavage (26)	Unknown	Late
A224L	IAP apoptosis inhibitor (27)	Unknown	Late
QP383R	IFN inhibitor (28)	Unknown	Late
I73R	Z nucleic acid-binding protein (29)	Unknown	Early
I177L	Virulence factor (30)	Unknown	Late
F317L	NF-kB inhibitor (31)	Unknown	Late
C717R	Uncharacterized	Unknown	Late
H339R	Alpha-NAC-binding protein (32)	Unknown	Late
CP204L	p30 early protein	Unknown	Early
B119L	ALR/ERV-like region	Unknown	Late
K145R	Major cytoplasmic protein (33)	Unknown	Late
K421R	Uncharacterized	Unknown	Late
C257L	Uncharacterized	Unknown	Late
H171R	Uncharacterized	Unknown	Late
H124R	Uncharacterized	Unknown	Late
M448R	Uncharacterized	Unknown	Early
H108R	Uncharacterized	Unknown	Late
E120R	Virus transport and IFN inhibitor (34, 35)	Unknown	Late
E423R	Uncharacterized	Unknown	Late
			

^*a*^ The first column shows the protein name. The second column shows the function, and the third column shows the location in the virion determined experimentally or predicted from the sequence. The fourth column shows if transcripts are detected predominantly early, late, or not confirmed (NC) (36). Other references are shown in the text or in the figure.

The architecture of the ASFV virion, determined by cryogenic-electron microscopy (6–8), revealed previously unknown features. The capsid was resolved to 4.1 Angstroms. The major capsid protein p72 consists of a double jelly fold resembling many other virus capsid proteins and is assembled as a trimer (37). A virus-encoded chaperone, pB602L, is required for p72 assembly but is not packaged in the virion (38). The capsid contains 8,280 major capsid protein p72 (B646L) copies organized into penta-symmetrons and tri-symmetrons. A network of four minor capsid proteins—pH240R, p49 (B438L), p17 (D117L), and M1249L—is also present and guides assembly. Each vertex appears to be formed by a penton complex composed of five copies of the protein pH240R (60 copies per capsid), which appear to be stacked over five copies of the protein p49 (7). The p49 protein appears to interact with immature inner virus membrane complexes. The p17 (pD117L) transmembrane protein is inserted in the inner virus envelope and links this with the p72 proteins in the outer capsid. The p17 protein was previously shown to be essential for the progression of membrane precursors to icosahedral structures (39). The large M1249L protein runs between each edge of the tri-symmetrons that bridge neighboring penta-symmetrons. This protein is proposed to be similar in function to the “tape measure” proteins encoded by other icosahedral nucleocytoplasmic large DNA viruses (6, 7, 40).

Proteomic analysis showed that nine other virion proteins contain one or two transmembrane domains and are presumed to be present in the inner viral membrane. Two proteins in the inner membrane, pO61L and p54 (*E183L*), are known to be involved in virus binding and entry into cells. The p54 protein is critical for the transformation and retention of modified host endoplasmic reticulum (ER) membranes in the virus factories (18). Two other membrane proteins, pE199L and pEP248R, are known components of a viral entry fusion complex involved in the fusion of the viral inner envelope with late endosomal membranes to release the virus core to the cytoplasm (14, 41). Entry fusion complexes in other giant viruses and vaccinia viruses contain similar conserved proteins. It has not yet been determined if other ASFV proteins are required for the fusion of the inner virus envelope with the endosomal membrane to allow entry of virus cores in the cytoplasm. The vaccinia virus entry fusion complex contains nine additional proteins (42). Thus, it seems likely that more ASFV proteins will be required for this process. As shown in Table 1, several ASFV virion particle proteins have transmembrane domains. Some of these may be involved in entry fusion. Two candidate proteins for these roles are pB169L and pB117L. The pB169L protein contains two transmembrane domains and has pore-forming activity (17). The pB117L membrane protein has low pH membrane permeabilizing activity (16). Another small protein, pEP84R, that is embedded in the virion’s inner membrane, is required to correctly target the core shell polyproteins and maintain the contact between the core shell and inner membrane (19). The proposed topology of EP84R makes it less likely that this protein is involved in the entry fusion process.

The core shell was resolved at low resolution (>8 angstroms) as an icosahedral structure. This shell is derived from two viral polyproteins pp220 and pp62, which are processed to individual proteins p150, p34, p37, p14, p5, p35, p15, and p8, respectively, by a virus-encoded Sumo-like protease, pS273R, that is also present in the core shell (20, 21). The viral genome and enzymes and factors required for the early stages of virus replication are packaged in the viral core. These include the eight subunits of the core virus-encoded RNA polymerase, two components of the putative early transcription factor, and enzymes involved in mRNA polyadenylation and capping. The cryo-EM structure of the multi-subunit RNAP was recently determined from *in vitro* expressed RNAP subunits (43) and from infected cell extracts. A major surprise was the co-purification of the M1249L minor capsid “tape-measure” protein with RNAP from infected cells. Thus, this very large M1249L protein is presumed to have roles in both capsid assembly and transcription regulation. Structures of the M1249L protein in the capsid and associated with RNAP are in different conformations, presumably mediated by interactions with other virus proteins (10, 11). Additional proteins identified in the virion proteome—and presumed to be in the virus core—include DNA-binding proteins such as the histone-like DNA-binding protein pA104R (44) and K78R. Components of the ASFV DNA base excision repair (BER) system are also packaged (9). This BER system may facilitate virus replication in the hostile oxidizing environment of the macrophage cytoplasm. Packaging in the virus core may make these proteins more readily available to repair virus DNA before the onset of DNA replication. Several proteins with roles in immune evasion were identified in the virion proteome (see section below). These included the pA224L IAP apoptosis inhibitory protein, pI73R Z nucleic acid binding protein, pC129R cyclic GMP-AMP (cGAMP) cleavage protein, and additional proteins shown to have IFN inhibitory properties including QP383R, pA137R, and pF317L or to act as virulence factors including pI117L.

This new knowledge on the virus structure and protein content has advanced knowledge on mechanisms of virus entry and assembly, providing new targets for intervention. The native structure of the major capsid protein has aided the mapping of epitopes for the design of diagnostic tests and interaction sites with host receptors. These may be targets for virus neutralization. For example, cryo-EM constructions of binding sites for five antibodies to p72 identified a major antigenic supersite. In initial studies, partial inhibition of virus infection was obtained using several antibodies that bind to this site (45). Additionally, virion proteins that are packaged, but not architectural, are likely to have roles early during entry and replication. Further research on these proteins is warranted to better understand the virus replication cycle and host interactions.

## VIRUS GENOME

The large linear double-stranded ASFV genome contains covalently cross-linked termini and adjacent tandem repeats. In this respect, it resembles the genomes of Poxviruses. Like poxviruses, ASFV forms head-to-head concatemers during DNA replication (4). The coding region ranges in size between 170 and 193 kbp. Open reading frames (ORFs), coding for at least 60 amino acids and not substantially overlapping with other ORFs, were originally annotated. Recent interrogation of the virus transcriptome by sequencing the 5′ end of mRNAs identified several highly transcribed ORFs, some of which were shorter and started within larger ORFs (36). Regions close to genome termini code for five different related multigene families (*MGF 100*, *110*, *300*, *360*, and *505*) that have evolved by a process of gene duplication or deletion and sequence transpositions between genome ends. These genome regions are most variable in length between isolates due to the gain or loss of members of these *MGF* genes as well as some sequence transpositions from one genome end to the other (4). As discussed below in “Virus evasion of host defenses,” many of these *MGF* genes are now known to code for proteins that inhibit innate immunity and can be associated with virulence in domestic pigs. An additional duplicated gene family comprises up to three members, including an early expressed membrane protein pKP177R (also designated p22).

Sequencing of a fragment of the gene coding for the major capsid protein p72 (B646L) has classified ASFV isolates into at least 23 genotypes that are present in Africa. This reflects long-term evolution within the sylvatic cycle in East Africa. Only genotypes I and II have spread out of Africa. The virulent pandemic genotype II strain was the only strain reported in China until a low-virulence genotype I strain was identified in domestic pigs (46). Subsequently, a virulent recombinant genotype II genotype I hybrid was identified first in China, then in Vietnam and Russia (47, 48). Since this recombinant had recently emerged, break points could be mapped to distinguish a mosaic of multiple fragments from each genotype distributed across the genome. Infection of the same cells by two different viruses is required for recombination to occur. Several types of viruses, including Poxviruses, have mechanisms to limit superinfection of cells by another strain. This has not yet been investigated for ASFV. Recombination between ASFV strains may occur during replication in hosts but also possibly in cell culture. This process is likely to be important during virus evolution, but further research is required to understand the mechanisms and frequency of recombination.

As sequences of more full-length genomes have become available, detailed bioinformatic comparisons between strains and genotypes have been applied. A machine-learning approach identified seven biotypes among the proteome sequences from 220 isolates (49). The availability of whole-genome sequences from several genotypes has provided the information to investigate epitopes and proteins important for inducing protection against challenge. Detailed knowledge of mechanisms and drivers for ASFV genome evolution is required to follow the virus spread and help predict the phenotype. This will be especially important for the design of vaccination campaigns.

## VIRUS REPLICATION

**Fig 2:**
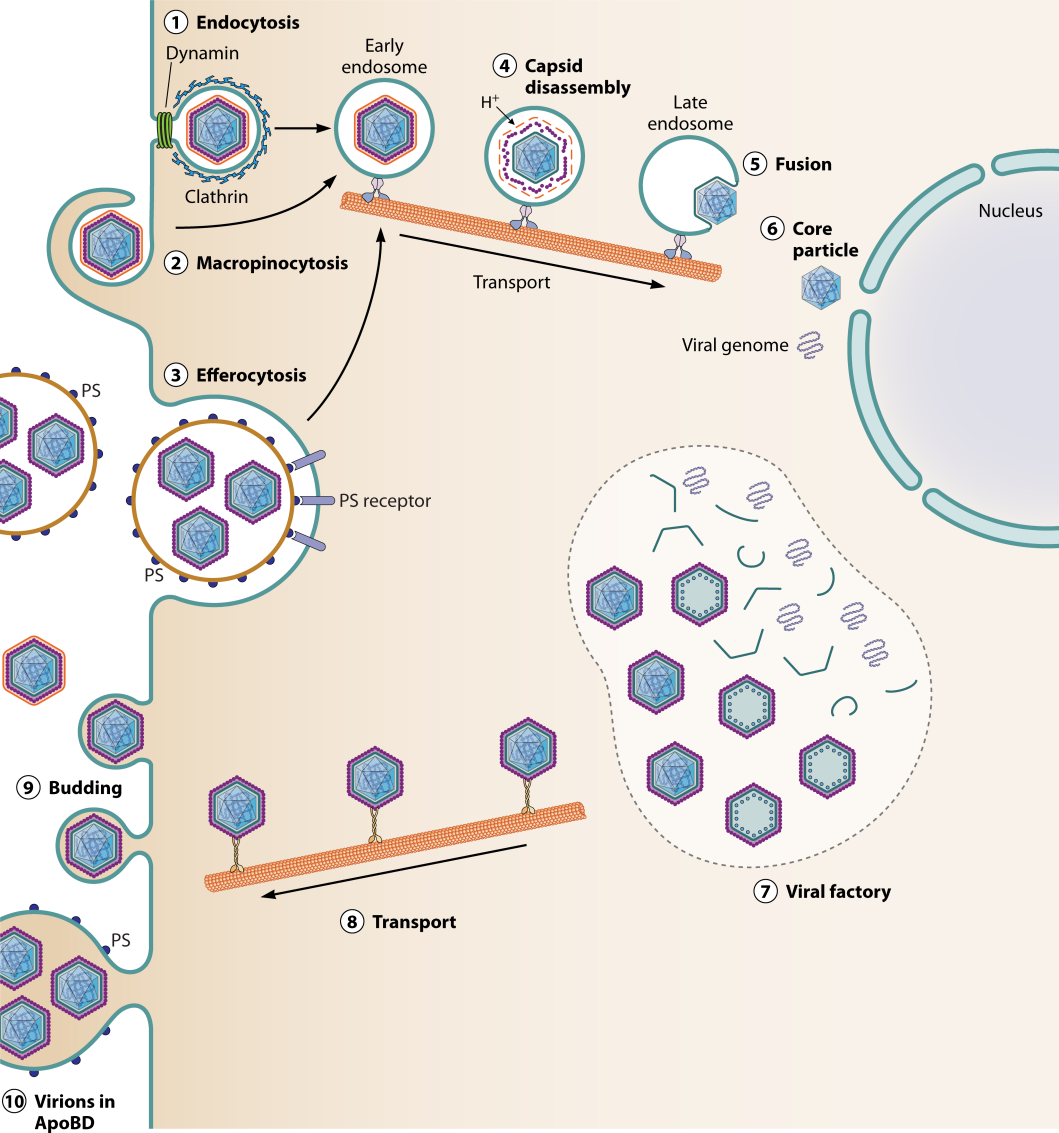
The ASFV replication cycle. The diagram shows the steps in ASFV replication numbered and described below and in the text. Virus particles enter cells by clathrin-mediated endocytosis (1), macropinocytosis (2), or efferocytosis (3) and are transported in endosomes via microtubules to perinuclear areas. The acidic condition in late endosomes leads to the disruption of the capsid (4), and the inner virus membrane fuses with the host late endosome membrane (5), releasing the virus cores into the cytoplasm (6). Early transcription takes place in partially uncoated cores in the cytoplasm. These codes for proteins are required for later replication. Following the onset of DNA replication in the cytoplasm, the pattern of gene transcription changes. The late transcripts code for proteins including those required for virus morphogenesis in the perinuclear factories (7). Host endoplasmic reticulum is modified by the insertion of virus proteins to form the virus inner membrane onto which the capsid is assembled. The core shell assembles inside the inner membrane, and the nucleoid components, including the DNA genome and proteins, are packaged inside the core shell. Mature intracellular virions are transported on microtubules (8) to the cell surface where they bud through the plasma membrane gaining the external membrane (9). Alternatively, intracellular virions can be released in apoptotic bodies (10).

The natural target cells for virus replication are porcine cells of monocyte/macrophage lineage at an intermediate to late stage of differentiation (4). However, the factors that restrict the ASFV tropism to these cell subsets are not well understood. ASFV can enter cells by several routes (see Fig. 2), including clathrin-mediated endocytosis and macropinocytosis (4). Recently, ASFV entry into macrophages by apoptotic mimicry was described (50, 51). Many viruses, including ASFV, have mechanisms to inhibit cell death by apoptosis to enable the replication of virions. Despite this, at late times of ASFV infection, apoptosis is observed in infected cells. Induction of apoptosis results in flipping of phosphatidylserine onto the outer layer of the plasma membrane. From this location, phosphatidyl serine can be incorporated into the external membranes of virions or membranes of apoptotic bodies as they bleb from the plasma membrane. Apoptotic bodies were observed to contain intracellular single membranes containing ASF virions. Extracellular ASF virions, or apoptotic bodies containing intracellular ASFV, can therefore bind to phosphatidyl serine receptors on macrophages, providing an additional route for virus entry. This route of ASFV entry was confirmed to be important since binding of Annexin V to block the binding of phosphatidyl serine to its cell surface receptors inhibited virus infection (50–52). Deletion of the A179L gene, which codes for an anti-apoptotic Bcl 2 family member, resulted in earlier apoptosis in infected porcine bone marrow (PBM) cells and reduced virus replication in cells. However, an increase in infection rate and earlier expression of ASFV genes was observed following infection of PBMs with the A179L gene-deleted virus. This was blocked by the addition of annexin V, confirming a role for surface phosphatidyl serine in increasing ASFV entry to macrophages (52). The induction of apoptosis at a late stage of infection could facilitate the spread of the virus to uninfected macrophages. ASFV manipulation of apoptosis may provide additional advantages for the virus in addition to facilitating spread. In other systems, the binding of phosphatidyl serine to its receptors has been shown to activate anti-inflammatory host responses (53). This may dampen the host’s proinflammatory response, thereby reducing virus clearance.

The entry of ASFV into macrophages by several routes explains why specific cell surface receptors for virus entry at the plasma membrane have not been identified. It also explains why neutralization by antibodies is not fully effective. However, it is not known which mechanisms are most important for ASFV entry and spread in *vivo*.

These diverse ASFV cell entry mechanisms all deliver virus to the endosomal pathway. The acidic condition in late endosomes facilitates the loss of the virus external envelope and icosahedral capsid. As discussed in the virus structure section, entry of the core particle to the cytoplasm occurs following the fusion of the virus internal envelope with that of late endosomes (41).

Recently, several host endosomal proteins that are important for virus entry have been identified. One of these was identified by targeted screening of endosomal proteins known to be important for the entry of other enveloped viruses. This screen identified the Niemann-Pick V type 1 (NPC1) as being important for ASFV entry (54). Two other studies used genome-wide CRISPR Cas9 screens to identify host genes in the WSL wild boar lung cell line that are important for ASFV replication (55, 56). Both identified genes coding for proteins in late endosome membranes. One study identified genes belonging to the swine leukocyte antigen complex II. The genes identified included MHC II-specific transcription factors RFXAP and CIITA, as well as SLA_DMA and SLA_DMB, components of the non-classical MHC II molecule SLADM. Knockout of these genes in WSL cells led to severe virus replication defects (55). In the second study, the authors identified the transmembrane protein 239 (TMEM239) as important for ASFV entry into cells (56). TMEM239 gene knockout pigs were generated. The replication of ASFV in peripheral blood mononuclear cells from the knockout pigs was significantly reduced, confirming TMEM239 as important for virus replication (56). The extension of similar knockdown approaches to natural porcine macrophage target cells will reveal further host pathways and molecules required for efficient ASFV replication. Comparison of these molecules in other susceptible hosts would be interesting to define conserved host proteins required for virus entry.

ASFV particles are transported in endosomes by the microtubule network to perinuclear areas, which are adjacent to the microtubule-organizing center. Here, core particles enter the cytoplasm, and ASFV early gene transcription begins in partially uncoated cores using the packaged virus-encoded transcription machinery, including RNAP, early transcription factors, and enzymes required for capping and polyadenylation of mRNAs. Host RNA polymerase II is not required for ASFV gene transcription. However, early studies suggested that the host nucleus is required for the replication of a tissue-culture-adapted ASFV strain, BA71V, in Vero cells. Discovery of ASFV DNA in the nucleus suggested that an early phase of DNA replication may take place in the nucleus. However, the potential role of a nuclear phase in ASFV replication is still unclear as the virus encodes the enzymes required for replication in the cytoplasm (57, 58). Following the onset of virus DNA replication in the cytoplasmic factories, a late gene transcription phase begins using a different set of transcription factors. These have not been functionally characterized, although candidates can be predicted by comparing sequences with vaccinia virus late transcription factors. An intermediate phase of virus gene transcription has been proposed, but the factors involved have not yet been characterized. Precise mapping of the 5′ transcription start sites and termination sites at different stages post-infection has enabled more accurate early and late gene transcription profiles to be determined (36, 59, 60). In general, early genes code for proteins required for the later phase of replication. Many important immune evasion genes, including almost all the MGF family genes, are also transcribed early. Late transcripts code for proteins required for virus assembly, including those packaged in virions to initiate the next round of replication, as well as proteins involved in host cell modulation or virus spread.

Intracellular single membranes containing virions that contain the nucleoprotein core are considered mature since they are infectious. The mechanism for selective packaging of components in the core is not understood. The intracellular mature virions are transported from virus factories on the microtubule network to the plasma membrane where they bud from the plasma membrane gaining an external envelope. Both intracellular mature and extracellular virions are infectious (4). As discussed above, intracellular virions can also be released from infected cells in apoptotic bodies.

Identifying proteins required for virus replication provides potential targets for intervention strategies including antivirals or antibodies. For example, the expression of essential ASFV genes in a helper cell line could allow a virus with the gene deleted to be grown and used to infect animals, causing an abortive replication that induces immune responses. Potentially, this could generate safer non-replicating vaccines. Knockout of host genes that are essential for virus replication could, in the long term, enable the development of transgenic pigs that are resistant to or can tolerate ASFV infection without developing clinical signs. Knowledge of essential virus-host protein interactions or the structures of enzymes or other factors that are essential for replication can facilitate the selection and design of antiviral compounds.

## VIRUS EVASION OF HOST DEFENSES

Very significant advances have been made in understanding how ASFV modulates and evades the host’s defenses to facilitate virus replication and survival. The impact of individual or multiple gene deletions on virus pathogenesis and induction of protective immune responses in pigs has identified genes important for virulence (see Table 2). In turn, this information has been used to develop rationally attenuated, gene-deleted ASFV live attenuated vaccines. This topic has been the subject of recent reviews by several authors (61–63). Here, additional information on the timing of expression and other known functions of virus evasion proteins and virulence factors is summarized (see Table 2), and the modulation of additional host pathways is discussed. The impact of individual ASFV proteins on host responses can be difficult to assess, particularly if they play an essential role during replication. Proteins that are expressed early during infection are more likely to have an important role in modulating host responses. Those expressed late during replication may have a more transient role. Host evasion proteins that are packaged in virus particles may have a role early during virus replication after release from virus core particles.

**TABLE 2:** ASFV encoded immune evasion proteins and virulence factors^*a*^

ASFV protein	Target	Function	RNA	Deleted		Attenuated	
				Single	Combined	Single	Combined
Type I IFN induction							
QP383R	Cyclic GMP-AMP synthase (28)	PLP-dependent transferase	Late	Yes	QP507L		Yes (64)
EP364R	cGAMP (26)	ERCC4 domain endonuclease	Late	No			
C129R	cGAMP (26)		Late	No			
B175L	cGAMP stimulator of interferon genes (STING) (65)	Late transcription factor	Late	No			
B318L	STING and IFNR1/2 (66)	Geranyl geranyl pyrophosphate synthase	Late	Yes		Yes (66)	
E184L	STING (67)		Late	Yes		Yes (67, 68)	
DP71L	STING (69)		Late	Yes	DP48R and DP96R		Yes (70)
H240R	STING Nemo IFNR1 and IFNR2 (12, 71)	Penton protein capsid	Late	Yes	MGF505-7R	Yes (72)	Yes (73)
l83L	IL-1β and STING (74, 75)	Binds IL-1β	Early	Yes		No (74)	
MGF360-12L	Type I IFN, NF-κB, and JAK/STAT (76)		Early	Yes	Several MGF360 and MGF505		Yes (77, 78)
MGF 360–13L	STING (79)		Early	No	Several MGF 360 and 505		Yes (77, 78)
MGF 505–6R	STING (80)		Early	No			
MGF505-7R	STING (81) IRF7, TBK1 (82), and IRF9 (83)		Early	Yes	H240R or MGF360-9L and MGF110-9L	Yes (84)	Yes (73, 85, 86)
MGF 505–11R	STING (87)		Early	No			
MGF505-2R	STING (88)		Early	Yes	Several MGF360 MGF505	Yes	
D117L, p17	STING/TBK1 (89)	Virus inner membrane	Late	No			
I267L	RIG-I (90)	RING finger containing	Early	Yes		Yes (90)	
A137R	TBK (24)	Virion protein	Late	Yes		Yes (91)	
A151R	TBK (92)	CXXC-motif	Early	Yes		Yes (93)	
DP96R	IRF3/TBK (94, 95)		Early	Yes	With UK or DP71L and A238L	Yes (96)	Yes (70, 97)
I215L	TBK1 and IRF9 (98)	E2 ubiquitin ligase	Early	No			
MGF360-11L	TBK1 (99)		Early	No	Several MGF360 MGF505		Yes (78)
MGF110-9L (with MGF 505–7R)	TBK1 (86)		Early	Yes	MGF360-9L MGF505-7R		Yes (100, 101)
D129L	IRF3/p300 (102)		Late	No			
DP96R	IRF3 (94)		Early	Yes	DP148R and DP71, B119L or CD2v		Yes (70, 97, 103)
E120R	TBK1/IRF3 (34)	Virion transport (35)	Late	No			
E301R	IRF3 (104)	PCNA DNA clamp (105)	Late	No			
I226R	IRF3/Nemo (106)		Late	Yes		Yes (107)	
M1249L	TBK1/IRF3 (108)	Capsid structure and transcription (7, 10, 11)	Late	No			
MGF360-4L	IRF3 (109)		Early	No			
MGF360-14L	IRF3 (110)		Early	No	Several MGF360 o MGF505		Yes (77, 78)
IFN signaling							
B318L	IFNAR1 IFNAR2 (66)	Geranyl geranyl pyrophosphate synthase	Late	Yes		Yes (66)	
l7L	STAT1 (111)		ND	Yes	l7L , l8L, l9L, l10L, and l11L	Yes (111)	Yes (112, 113)
B475L	STAT2 (114)		Late	No			
I215L	IRF9 (98)	E2 ubiquitin ligase	Late	No			
MGF360-10L	JAK1 (70)		Early	Yes	Several MGF360 MGF505		Yes (77, 78)
MGF360-9L	STAT1 and STAT2 (115)		Early	Yes	Several MGF360 or MGF505		Yes (77, 78)
MGF505 7R	IRF9 (82, 83) JAK1- and JAK2- (116)		Early	Yes	H240R or MGF3609, MGF110-9L	Yes (84)	Yes (73, 85, 86)
S273R	STAT2 (117)	Sumo-like protease	Late	No			
K205R	IFNAR1/2 (118)		Early	No			
NF-κB activation							
A238L	NF-κB/p300 (119)		Early	Yes	EP402R		Yes (120, 121)
A528R	P65 (122)		Early	No			
D345L	IKK-β kinase (123)	Lambda nuclease	Late				
I215L	IRF9, STAT2 (98, 124)	E2 ubiquitin ligase	Early	No			
F317L	IKK-β (31)		Late	No			
MGF300-2R	IKK-α and IKK-β (125)		Early	Yes		Yes (125)	
MGF300-4L	IKK-a and IKK-b (126)		Early	Yes		Yes (126)	
Inflammasome							
MGF505-7R	NLRP3 (84)		Early	Yes		Yes (84)	
S273R	Gasdermin D (127)		Late	No			
l83L	IL1-β (74)			Yes		No (74)	
Apoptosis							
A179L	Bcl 2 family (128, 129)		Early	Yes		Yes (52)	
A224L	IAP family (27)		Late	Yes		No (130)	
EP153R	p53 (131)		Early		DP148R and K145R		Yes (132)
DP71L	eIF-2α CHOP/ATF4 (133)		Late	Yes	DP148R and DP96R		Yes (70)
Adhesion proteins							
CD2v	Red blood cell binding		late	yes	With EP153R and A238L	No	Yes (120, 121)
Other functions							
I117L			Late	Yes	CD2v or MG F110-5L-6L	Yes (30)	Yes (134, 135)
I73R	Z-α domain (29, 136)		Early	Yes		Yes (136)	
A104R	DNA binding		Late	Yes		Yes (137)	
E111R			Late	Yes		Yes/no (138, 139)	

^*a*^ The first column shows the ASFV protein name and host pathway targeted. The second column shows the host protein or complex targeted. The third column shows functions determined experimentally or predicted from the sequence. The fourth column shows the main transcription phase early or late (36). The fifth column indicates if the gene has been deleted from the ASFV genome singly or in combination with other genes. The sixth column indicates if the single or combined gene deletion is attenuated in pigs. References are provided in the table and in the manuscript text.

### Interferon induction and responses

The type I IFN pathway is recognized to be the major host antiviral pathway. Type I IFNs are secreted from infected cells and signal through cell surface IFN receptors to activate JAK/STAT signaling pathways in bystander or infected cells. This results in transcriptional activation of hundreds of IFN-stimulated genes (ISGs). These ISGs have diverse roles, including as host resistance factors limiting virus replication, in activation of host innate and adaptive immune responses, and in feedback loops to further activate or suppress the type I IFN response. The type I IFN response is activated when host pattern recognition receptors (PRRs) recognize pathogen-associated molecular patterns (PAMPs). Nucleic acids, either DNA or RNA, are the main PAMPs detected during virus infections. The cytosolic DNA detector, cyclic GMP-AMP synthase (cGAS), binds to DNA catalyzing the production of 2′−3′ cGAMP. cGAMP activates STING (stimulator of interferon genes), inducing its translocation from the endoplasmic reticulum to the trans-Golgi network. Here, STING acts as a scaffold to recruit TANK-Binding Kinase 1 (TBK1) and Interferon Regulatory Protein 3 (IRF3). Phosphorylated IRF3 dimers translocate to the nucleus, activating the IFN-beta promoter to transcribe type I IFNs. Cytosolic viral RNA is sensed by RIG-I-like receptors. Their activation leads to interaction via CARD domains with the mitochondrial antiviral signaling protein (MAVS) (140, 141), which, in turn, relays the signal to TBK1 and activation of IRF3 as discussed above. Viral nucleic acids are also recognized by Toll-like receptors present in the cell surface or early endosomal membranes. The binding of nucleic acid to the external domain of Toll-like receptors (TLRs) results in the assembly of complexes containing Myd88/Trif at their cytoplasmic domain. These signals link to the TBK-1 complex to activate IRF3 and type I IFN gene transcription.

Many ASFV-encoded proteins have been shown to inhibit type I IFN induction (see Table 2). These act at multiple points in the pathway (61, 63). Since the ASFV DNA genome replicates in the cytoplasm, many studies have focused on identifying proteins that inhibit IFN induction by stimulation of the cGAS-STING pathway. Two proteins, pCP129R and pEP364R, act by promoting the degradation of cGAMP (26), and one protein, pQP383R, has been reported to inhibit cGAS enzymatic function by promoting its palmitoylation (28). Few studies have investigated the inhibition of IFN induction mediated by RIG-I receptors. The pI267L protein was shown to inhibit RIG-I receptor-mediated induction of type I IFN by disrupting Riplet-RIG-I interaction to impair Riplet-mediated K63 polyubiquitination and activation of RIG-I (90). Transcription of AT-rich double-stranded DNA mediated by cytoplasmic RNA polymerase III provides the RNA PAMPs to induce RIG-I activation. Overlapping transcripts from both DNA strands may also generate double-stranded DNA that can activate the RIG-I pathway.

Downstream signaling from these initial PAMP and PRR interactions activates the TBK-1 kinase and the IRF3 transcription factor. Several proteins, including MGF505 proteins, 6R, 7R, 11R, pMGF360-11R, have been reported to promote degradation of STING (80, 82, 87, 88). pE184L was shown to inhibit STING oligomerization (67). Several other proteins were shown to inhibit type I IFN induction by targeting TBK1 or IRF3. These include the E2 ubiquitin-conjugating enzyme pI215L (142), pMGF110-9L (143), pMGF505-3R, pDP96R (94), pMGF360-14L (110), pMGF360-4L (109), pI226R (106), pA151R (92), pH240R (12), pA137R (24), p17 (D117L) (144), pE120R (34), and pM1249L (108).

Inhibition of the IFN response pathway has been less studied, although several proteins that inhibit the JAK/STAT pathway have been identified. Activation of interferon response genes through the type I IFN receptor was shown to be inhibited by proteins pMGF505-9L, pB318L (66), pMGF360-9L (115), pMGF360-10L (145), pH240R, pA104R histone-like protein (146), and pS273R (117). The ASFV protein pl7L was shown to prevent phosphorylation and activation of STAT1, thus inhibiting IFN-γ-induced JAK-STAT signaling and suppressing the expression of IFN-γ-induced antiviral genes (111). The pK205R protein was shown to interact with the cytoplasmic domains of IFNAR 1 and IFNAR 2, preventing the activation of JAK/STAT signaling (118). pMGF505-7R was shown to inhibit signaling from the type II IFN receptor by an indirect mechanism (63).

### Cell death

An important mechanism by which the host controls virus infection is by inducing cell death by apoptosis to limit the production of infectious progeny virus. ASFV-encoded inhibitors of apoptosis include pA179L, an anti-apoptotic Bcl2 family member, that binds to several pro-apoptotic Bcl2 family members (128). The deletion of the A179L gene results in a large reduction of virus replication due to early induction of apoptosis (52). The pA224L is a member of the IAP family of anti-apoptotic proteins and binds to caspase 3, inhibiting its activity (27). pA224L is expressed late during infection and packaged in virus particles (130, 147). Other inhibitors of apoptosis act indirectly by inhibiting the transcription of pro-apoptotic genes or increasing the transcription of anti-apoptotic genes. The DP71L protein restores global protein synthesis by recruiting protein phosphatase 1 to dephosphorylate eIF-2α. Transcription and translation of the ATF4 and CHOP transcription factors, which activate pro-apoptotic genes, are thereby reduced (133). Deletion of the *DP71L* gene from a virulent ASFV isolate did not increase levels of phosphorylated eIF-2α, suggesting that the virus has other mechanisms to control this phosphorylation (133). pEP153R is expressed early and has been reported to inhibit p53-induced apoptosis (131). Inhibition of NF-κB activation can also reduce the expression of pro-apoptotic proteins such as c-FLIP.

### Inflammation

Induction of pro-inflammatory responses following infection is important for the activation of host innate and adaptive immune responses to control virus infection. Mechanisms by which ASFV controls the inflammatory response include inhibiting the signaling pathway that activates the NF-κB transcription factor. NF-κB induces the transcription of hundreds of genes, including those coding for proinflammatory proteins. NF-kB is held in the cytoplasm in an inactive complex with IkB-α. The activation of IKK kinases results in phosphorylation and ubiquitin-mediated degradation of IKB-α, thus releasing NF-κB to translocate to the nucleus and bind to promoters containing NF-κB-binding elements. ASFV pA238L protein was the first protein shown to inhibit the host cell inflammatory responses. pA238L acts by inhibiting recruitment of the p300 transcriptional co-activator to specific enhanceosomes, including those formed on TNF-α and cyclooxygenase-2 promoters, during ASFV infection (148). Subsequently, several ASFV proteins have been demonstrated to inhibit the activation of NF-κB. These include pI215L (149), pMGF505-5R/6R (122), pD345L (123), pF317L (31), pMGF300-2R (150), pMGF300-4L (126), and pS273R (117). These act at the level of the IKK kinase or IK-β. Among these NF-κB inhibitory proteins, pD345L contains a YqaJ-like viral recombinase domain, pI215L is an E2 ubiquitin-conjugating enzyme, and as expected, it targets several pathways.

Additionally, ASFV has mechanisms to inhibit the formation and activation of the inflammasome, thus inhibiting the processing and release of pro-inflammatory cytokine IL-1β. ASFV pMGF505-7R and pH240R interact with NLRP3 to inhibit ASC oligomerization, thereby inhibiting caspase-1 activation and IL-1β secretion (81, 84). Furthermore, the pL83L protein was shown to bind to pro-IL1-β, preventing its processing and secretion (74). The pS273R protease is known to process the viral polyproteins pp220 and pp62 as well as several host proteins. For example, pS273R cleaves the N-terminal fragment of Gasdermin D, inhibiting its activity to prevent pyroptosis and release of IL1-β (127).

### ASFV modulation of ER stress and protein translation

ASFV recruits ER membranes to virus factories, and these are modified by the insertion of virus proteins and incorporated into the virion inner membranes. This can induce an unfolded protein response (UPR), leading to the activation of the PERK-eIF-2α, ATF4, ATF6, and IRE-1-XBP1 pathways. In macrophages infected with wild-type virulent ASFV, the UPR was shown to be triggered and resulted in Ca ^2+^ release from the ER via the inositol triphosphate receptor. The ATF6 pathway has been shown to be activated following ASFV infection, resulting in the transcriptional activation of many genes, including those of chaperones to alleviate protein misfolding. Knockdown, using small interfering RNAs (siRNAs), or drug inhibition of the ATF6 or IRE-1-XBP1 pathways impaired ASFV replication, showing these pathways are beneficial for replication. Activation of PERK kinase or the double-stranded RNA-activated PKR kinase results in phosphorylation of the translation initiation factor eIF-2α, leading to the shut-down of global protein synthesis while maintaining the translation of a subset of mRNAs, including ATF4 and CHOP. These factors activate the transcription of many genes, including those with pro-apoptotic functions. The ASFV protein pDP71L recruits protein phosphatase 1 to dephosphorylate eIF-2α, thus restoring global protein synthesis and enhancing virus replication. The pB117L is present in the virion inner membrane and has been shown to have viroporin-like activity, suggesting this protein may be involved in Ca2+ release and activating the ATF6 response (151). As predicted, ectopic expression of pB117L was shown to induce the UPR and activate an ATF6-dependent promoter, suggesting this is a mechanism for activation of the UPR during ASFV infection. Direct interaction of pB117L and ATF6 was confirmed. These results demonstrate how ASFV manipulates the UPR to facilitate virus replication (151). The formation of stress granules can regulate the host response to virus infection. During ASFV infection, the formation of stress granules is inhibited by cleavage of the nucleating protein G3BP1 (Ras-GTPase-activating protein [SH3 domain] binding protein 1) by the virus-encoded pS273R protease (152).

The formation of *Z*-nucleic acid during virus infections is an important signal that leads to the activation of host antiviral pathways. This is mediated by the binding of *Z*-nucleic acid to host protein sensors, including ZBP1 and ADAR1. The vaccinia virus E3L protein and fish cyprinid herpes virus 3 ORF112 proteins code for *Z*-nucleic acid-binding proteins. The E3L protein inhibits the interaction of *Z*-nucleic acid with ZBP1 proteins, thus preventing the activation of cell death by necroptosis (153). The ORF112 protein is proposed to sequester Z-RNA in ribonucleic acid-containing particles, thus preventing the interaction of Z-RNA with cellular dsRNA sensors (154).

The structure of the 73 amino acid pI73R ASFV protein showed that it had a similar structure to the *Z*-nucleic acid binding domain (*Z*-α), comprising the complete length of the protein. The protein pI73R binds to CpG repeat-containing DNA, which has a propensity to form *Z*-nucleic acid (29, 136). The protein was expressed abundantly from early times and packaged in virus particles, suggesting it has a role throughout infection. Deletion of the gene reduced virus replication and virulence in pigs (136).

The ASFV pCD2v protein is expressed on the surface of infected cells and extracellular virus particles and is required for binding to red blood cells. Residues required for this binding have been mapped on the CD2v IgG1 domain (155, 156). Expression of the pCD2v protein on the surface of virions dramatically prolongs the period of virus persistence in blood, thereby facilitating virus persistence and spread. Deletion of the ASFV genes coding for the pCD2v protein or the pEP153R C-type lectin domain-containing proteins alone did not reduce virulence; however, deleting both genes strongly attenuated the virus in pigs, suggesting a synergy in the function of the two proteins (157). This assertion may depend on the ASFV strain used. For example, deletion of the gene encoding pCD2v alone did attenuate the virulent strain BA71 (158).

### Immune evasion proteins: summary and future directions

Although much has been learned about how ASFV evades host innate immunity, many gaps in knowledge remain. Many inhibitors of type I interferon responses have been identified, but their relative impact alone or in combination with other inhibitors is poorly understood. In some studies, proteomics has been used to identify virus or host proteins that interact together and define interaction networks (159–161). Expansion of these studies would help define synergy between networks, aiding the prediction of the impact of individual proteins and pathways during infection. Some key host response pathways involved in the induction of innate and adaptive immune responses and pathogenesis have been little studied. These include both antigen processing and inflammation. Host restriction factors that limit virus replication are also poorly understood. Interactions between ASFV-infected cells and bystander cells play a key role in determining the outcome of infection, but they are poorly understood.

### Virulence determinants

The high level of redundancy in ASFV proteins that target the host type I interferon induction and response pathways indicates that multiple proteins may be required to block the pathway, and some proteins may function at different stages of replication. Functions, identified from initial experiments using ectopically expressed genes not in the context of infection, should be confirmed in ASFV-infected macrophages. The effects of gene knockouts, other mutations, or gene knockdowns can provide supporting evidence. Due to functional redundancy, phenotypic effects may not be observed from single gene deletions but could be observed if additional genes are deleted. Information on the cellular targets for immune evasion proteins is summarized in Table 2. The table also shows which genes have been deleted from the ASFV genome, either singly or in combination with other genes. These have been tested *in vitro* in cell culture and for some recombinants *in vivo* by infection of pigs.

Deletion of individual genes coding for inhibitors of IFN induction, which resulted in virus attenuation in pigs, included *B318L* (66), *MGF505-7R* (81), *H240R* (72, 162), *A137R* (91), I266R (107), *I267L* (90), *E184L* (68), *MGF505-2R* (88), *QP383* (in combination with *QP509L*) (64), and *A151R* (93). Deletion of the *B318*L gene, encoding a trans-geranylgeranyl-diphosphate synthase, negatively regulated cGAS-STING and IFNAR-JAK-STAT signaling pathways and reduced virus virulence (66). Likewise, the deletion of genes coding for proteins that inhibit IFN responses, such as *MGF505-7R* (116) and l7L (111), also attenuated the virus infection in pigs.

Deleting combinations of genes that target the type I IFN response was also successful in attenuating the virus. For example, deleting *DP96R* in combination with *UK*, *DP71L*, *A238L*, *DP148R*, and *DP71L* or *B119L* resulted in virus attenuation in pigs. However, the relative impact of each gene deletion is not clear. Larger deletions of several multigene family genes, including IFN inhibitory genes, also reduced virus virulence in pigs. A deletion of six MGF 360 or 505 genes (*MGF505-1R*, *MGF360-12L*, *MGF360-13L*, *MGF360-14L*, *MGF505-2R*, and *MGF505-3R*) attenuated virulent virus (77). Overlapping deletions of four genes identified two of these genes, *MGF360-12L* and *MGF505-1R*, as contributing most to the attenuation (78). Simultaneous deletion of 24 genes from near the left genome end, including members of multigene families *MGF110*, *MGF100*, *MGF360*, *MGF300*, and *MGF505*, was achieved and caused a 10-fold growth defect in macrophages (163). Virulence in pigs was not tested. Multiple gene deletions of MGF family genes have demonstrated complexities of interactions between the genes (78).

Deletion of genes that inhibited NF-κB showed that deletion of *MGF505-5R/6R*, *MGF300-2R*, and *MGF300-4L* deletion attenuates ASFV in pigs (125, 126). Deletion of gene *l10L* in combination with *l7L*, *l8L*, *l9L*, and *l11L* also attenuated virus (112). Likewise, deletion of genes that inhibited inflammasome activation or secretion of IL-1β also reduced virulence, for example, *MGF505-7R*. As this is a multi-functional protein, it is not clear through which pathway the deletion had the greatest effect. Deletion of the *H240R* gene increased inflammatory responses and reduced virulence of ASFV (162).

Of the genes coding for apoptosis inhibitors, deletion of the *A179L* anti-apoptotic Bcl2 protein family member greatly reduced virulence (52). Deletion of the IAP family member gene *A224L* (130) did not reduce virulence, whereas deletion of *DP71L* reduced virulence when deleted from some strains but not others (164). Deletion of *DP71L* in combination with *DP96R* and *DP148R* (70) achieved good levels of attenuation and protection against challenge.

Deletion of the genes for virion proteins *H240R* (72, 162) , *A137R* (91), *H108R* (165), or *I177L* (30) reduced virus virulence. Deletion of the histone-like protein gene *A104R* dramatically reduced virulence (137). Deletion of the ASFV genes coding for the pCD2v protein or pEP153R C-type lectin domain-containing proteins alone did not reduce virulence, but deleting both genes strongly attenuated the virus in pigs, suggesting a synergy in the function of the two proteins (157). Deletion of the gene coding for the *I73R* Z nucleic acid-binding protein strongly attenuated virus (136). Deletion of *MGF 110–9L* alone attenuated virus (166), and in combination with *MGF360-9L*, two viruses were highly attenuated in pigs RNA compared with parental ASFV.

Deletion of many genes (about 20 have been tested) singly did not reduce virus virulence. These genes included the *C962R* DNA primase (167) and enzymes in the base excision repair system, *E165R* dUTPase (168), *E296R* endonuclease (169), and *O164L* DNA polymerase X (170). A lack of the base excision repair system may lead to an increased mutation rate over time, which could affect replication rates.

### Virulence factors: summary and contribution to modified live vaccine development

The accumulation of knowledge on ASFV proteins that target activation of the host innate immune system and the impact of deleting those genes from the genome has provided much new information that has been used to develop candidate-modified live vaccines. The many *in vitro* studies of virus infection in macrophages show that virulent ASFV strongly suppresses type I IFN responses. Deletion of different combinations of virus genes that target this pathway, as expected, increases this response and can be correlated with virus attenuation in pigs and induction of a protective immune response. Deletion of some gene(s) reduces virus replication in cells and/or *in vivo* in pigs. This can result in a failure to induce a sufficiently high immune response in pigs to protect them from challenges with virulent viruses. In the future, improved understanding of how ASFV manipulates host responses, including inflammatory responses and antigen presentation, could enable safer and more effective modified live vaccines to be developed. For candidate vaccines to be considered for further development, initial experiments should demonstrate good levels of safety and efficacy across a dose range. In many current candidate vaccine strains, more than one gene has been deleted to achieve better safety and efficacy. In the future, predictions on the impact of multiple gene deletions may become more certain as more knowledge is gained on their predicted impact on host pathways and their relationship to virus virulence and induction of a protective immune response.

To date, only the single gene deletion of the *I177L* gene (30) or the multiple deletions of six *MGF360* and *MGF505* genes (77) from genotype II viruses have been taken forward for commercial production and further larger-scale testing in the field. In 2023, these were licensed for use in Vietnam. Other vaccine candidates will almost certainly be developed commercially. Challenges for the future will include the development of a serological diagnostic test to Distinguish Infected from Vaccinated Animals (DIVA). This will require deleting gene(s) coding for one or more immunogenic proteins. Potential DIVA targets identified include genes coding for proteins pEP153R, pCD2v, and pA137R (134, 171, 172).
